# Shielding the Nerve: A Systematic Review of Nerve Wrapping to Prevent Adhesions in the Rat Sciatic Nerve Model

**DOI:** 10.3390/jpm13101431

**Published:** 2023-09-24

**Authors:** Maximilian Mayrhofer-Schmid, Tess T. Klemm, Martin Aman, Ulrich Kneser, Kyle R. Eberlin, Leila Harhaus, Arne H. Boecker

**Affiliations:** 1 Department of Hand-, Plastic and Reconstructive Surgery, Burn Center, BG Trauma Center Ludwigshafen, Department of Hand- and Plastic Surgery, University of Heidelberg, Ludwig-Guttmann-Str. 13, 67071 Ludwigshafen, Germany; 2 Hand and Arm Center, Department of Orthopaedic Surgery, Massachusetts General Hospital, Harvard Medical School, Boston, MA 02114, USA; 3 Division of Plastic and Reconstructive Surgery, Massachusetts General Hospital, Harvard Medical School, Boston, MA 02114, USA; 4 Department of Hand Surgery, Peripheral Nerve Surgery and Rehabilitation, BG Trauma Center Ludwigshafen, 67071 Ludwigshafen, Germany

**Keywords:** peripheral nerve injuries, biomaterials, microsurgery, nerve regeneration, nerve scarring, nerve adhesions

## Abstract

Background: Peripheral nerve pathology is frequently encountered in clinical practice among peripheral nerve and extremity surgeons. One major factor limiting nerve regeneration and possibly leading to revision surgeries is the development of traumatic or postoperative adhesions and scarring around nerves. In experimental models, different materials have been studied to limit scar tissue formation when wrapped around nerves. Methods: A systematic review of studies describing nerve-wrapping materials in a non-transectional rat sciatic nerve model was performed following the PRISMA guidelines. Literature describing nerve-wrapping methods for the prevention of peripheral nerve scarring in rat sciatic nerve models was identified using PubMed and Web of Science, scanned for relevance and analyzed. Results: A total of 15 original articles describing 23 different materials or material combinations for nerve wrapping were included. The heterogeneity of the methods used did not allow a meta-analysis, thus, a systematic review was performed. Out of 28 intervention groups, 21 demonstrated a preventive effect on scar tissue formation in at least one qualitative or quantitative assessment method. Conclusions: The analyzed literature describes a variety of materials from different origins to limit peripheral nerve scarring and adhesions. Thus, a scar-preventive effect by wrapping peripheral nerves as adhesion prophylaxis seems likely. However, a quantitative comparison of the studies to identify the optimal material or technique is not possible with the diversity of used models and study designs. Therefore, further research needs to be performed to identify the optimal nerve wraps to be used routinely in clinical practice.

## 1. Introduction

Peripheral nerve pathology is frequent and poses both a clinical and economic challenge [1]. Postoperative or traumatic scarring and adhesions around peripheral nerves can cause debilitating symptoms in affected patients, hindering regeneration and sometimes leading to further surgical treatment [2]. Scar development is a normal and essential part of regeneration after peripheral nerve injuries, stabilizing the wound and giving the necessary tissue structure for axonal sprouting [3]. However, extensive scarring and adhesions may compress the nerve, impair its essential gliding ability, and can result in fibrosis within the neural and perineural tissue [4]. This, in turn, leads to decreased nerve perfusion and impaired regeneration after nerve injury [5,6]. Nerve scarring and adhesions occur in the connective tissue around nerve fiber bundles, known as perineurium and epineurium, and in the connective tissue between the nerve and its surrounding tissues, labeled as paraneurium [7].

Besides neurolysis and occasionally the provision of additional soft tissue coverage, effective clinical options for the treatment of primary or secondary nerve scarring are limited [2,8]. However, since the first clinical reports of positive effects and improved outcomes after vein wrapping in the surgical treatment of extensive scarring around nerves, additional research has been performed in this field and further treatment options have been explored [9].

**Figure 1:**
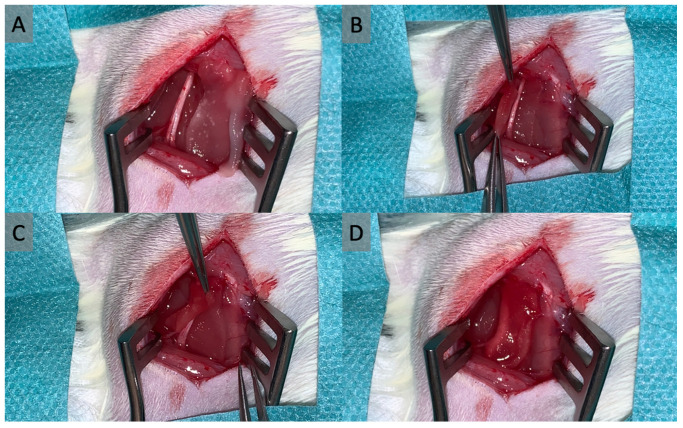
In this example for nerve wrapping, a collagen matrix is being wrapped around the sciatic nerve of a rat to limit scar tissue formation. The matrix is initially placed next to the nerve (**A**), then carefully pulled through underneath the nerve (**B**), and finally wrapped around it (**C**,**D**).

Numerous methods of wrapping with autologous or bioartificial materials have been described to improve peripheral nerve regeneration and prevent scar tissue formation. The underlying idea of this concept is to create a barrier around the nerve which prevents the formation of extensive scarring and adhesions between the nerve and its surrounding tissue and preserves the gliding ability of the nerve while allowing the diffusion of nutrients to the nerve [10]. However, assessing the effects of the material used is challenging when distinguishing between direct positive influences on nerve regeneration and secondary positive influences due to limited or prevented scarring. Especially the wrapping of peripheral nerves with a spacer material placed between the nerve and the surrounding tissue seems to provide favorable results in limiting peripheral nerve scarring [10]. One example of this technique is shown in Figure 1, where a synthetic collagen matrix is being wrapped around a part of a rat’s sciatic nerve.

While the rat sciatic nerve model is the most popular animal model for peripheral nerve injury and scarring, various methods are described to induce peripheral nerve scarring and evaluate outcomes in an experimental setting [11]. A number of studies use transection and consecutive epineural sutures for scar induction, while some authors use less traumatic methods avoiding neurotmesis [11]. However, transection injuries are significantly more traumatic than other methods of scar induction, comparing materials tested with different injury types is hardly possible.

This study describes and reviews previously explored methods for nerve wrapping to prevent scarring around peripheral nerves in the rat sciatic nerve model after peripheral nerve scarring induction without neurotmesis.

## 2. Methods

This review was performed in accordance with the “Preferred Reporting Items for Systematic reviews and Meta-Analyses” (PRISMA) guidelines to ensure transparency and reproducibility [12].

### 2.1. Search Strategy

The scientific databases MEDLINE using the PubMed^®^ interface and Web of Science^TM^ were used to identify publications matching the search query “(rat) AND ((peripheral nerve) OR (sciatic nerve)) AND ((wrap) OR (wrapping) OR (cover) OR (covering) OR (coat) OR (coating) OR (barrier) OR (space) OR (spacer)) AND ((injury) OR (scar) OR (adhesion) NOT (transection) NOT (cut))”. The search was completed on 30 September 2022. The reference lists of studies included in the full-text screening were also searched and 10 additional publications were identified (citation search).

### 2.2. Inclusion Criteria

For this systematic review, the inclusion criteria were (1) a rat sciatic model inducing peripheral nerve scarring and perineural adhesions, (2) nerve coating/wrapping or any method to install a spacer material between the nerve and the surrounding tissue at the time of injury, (3) assessment of nerve adhesions/scar formation, and (4) available full text in English or German.

### 2.3. Exclusion Criteria

The review excluded studies matching the following criteria (1) nerve cut or transection injury, (2) other animal species than the rat, (3) non-sciatic nerve models, (4) in-vitro models, (5) other treatment/prevention methods than spacer material application, (6) no negative control group (defined as animals with injury but without spacer application), (7) study type of review/meta-analysis, (8) language other than English or German, and (9) publication date after September 2022.

### 2.4. Study Selection

**Figure 2:**
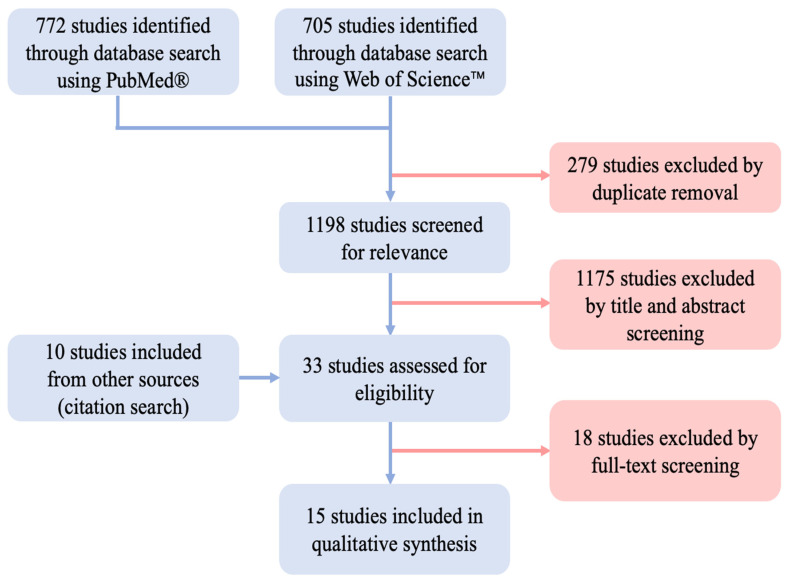
Flow diagram illustrating the study screening and selection process performed according to the PRISMA guidelines [12].

Two independent reviewers scanned the identified studies. First, titles and abstracts were screened for each included study. Next, full texts were screened for all studies not excluded in the abstract screening. In case of disagreement, a consensus decision was made with the support of a senior reviewer. The process is depicted as a flow diagram in Figure 2.

### 2.5. Data Extraction

The publications included in the final review were analyzed and their data were extracted and compiled for qualitative synthesis using Microsoft Excel Version 16.77.1 (Microsoft Corporation, Redmond, WA, USA). Tables for comparison of injury, intervention, assessment, follow-up time, and effect were used to estimate the trend of the results from the included studies. The used biomaterials were summarized regarding their regenerating qualities as a second objective. In studies with multiple intervention groups, including groups with transection injuries, only the groups matching our inclusion criteria were used for qualitative synthesis.

## 3. Results

### 3.1. Study Selection

Using PubMed^®^, 772 studies were identified and 705 studies were found using Web of Science^TM^. After excluding duplicates, 1198 studies remained. By applying the inclusion and exclusion criteria, 1175 studies were excluded during the title and abstract screening. By identifying studies from the references of the retrieved papers (citation search), 10 further studies were included for full-text assessment. Full-text assessment was performed on 33 studies in total. Of these, 15 studies met the final inclusion criteria and were used for qualitative synthesis [13,14,15,16,17,18,19,20,21,22,23,24,25,26,27].

### 3.2. Study Characteristics

In total, eight different methods of scarring induction were used in all included studies [13,14,15,16,17,18,19,20,21,22,23,24,25,26,27], and four studies used a combination of multiple methods [16,21,25,26]. Two studies compared the effects of their investigated prevention method on multiple scarring techniques [14,18]. Twenty-three different spacer materials and material combinations were investigated [13,14,15,16,17,18,19,20,21,22,23,24,25,26,27]. The follow-up time until the final scarring assessment ranged from 4 weeks to 5 months, with most studies evaluating at 6 weeks after the initial surgery [13,14,15,16,17,18,19,20,21,22,23,24,25,26,27]. In total, 475 sciatic nerves were assessed in all intervention groups, ranging from 7 to 42 per study, with a median of 15 assessed nerves per study group [13,14,15,16,17,18,19,20,21,22,23,24,25,26,27]. Basic study characteristics are shown in Table 1.

### 3.3. Scarring Assessment

Assessing the perineural scar formation with either qualitative or quantitative methods, 14 studies reported improved outcomes in at least one intervention group [13,14,15,16,17,18,19,20,21,22,23,24,26,27], and 1 study reported no significant difference in its only intervention group [25].

For scarring assessment, different approaches were used, including macroscopic evaluation (qualitative and quantitative), histological evaluation (qualitative and quantitative), and biomechanical testing (all quantitative). Eight studies reported quantified macroscopic scar assessment [14,15,17,19,20,23,24,27], in which twelve of nineteen intervention groups were reported to have significantly improved results after spacer application and the remaining seven not demonstrating a significant effect in this assessment method. All of these studies used the grading system for adhesions established by Petersen et al. [15], except for one study with three groups, where a modified system was used [17]. Using histological or microscopic methods, six studies with eleven groups combined reported quantified outcomes [15,20,23,24,25,27], of which eight intervention groups were significantly improved compared to their control groups. Biomechanical testing was performed in five studies with nine groups combined [13,17,18,19,21], in eight of which a significant improvement following spacer application was observed. Three studies used only descriptive methods for scarring assessment, all of which reported improved outcomes [16,22,26]. Assessment methods and overall outcome tendencies are shown in Table 2.

### 3.4. Used Spacer Materials

With 23 different spacer materials or material combinations investigated, this collective of studies documents various approaches for the spacer technique [14,15,16,17,18,19,20,21,22,23,24,25,26,27,28]. Classifying biomaterials in a systematic way is important to identify trends and potentials within groups of materials. In the literature examining biomaterials used as nerve conduits, these materials are frequently divided into two large groups: materials of natural and materials of synthetic origin. These are further divided into subgroups regarding the main components [29]. Since tissue transplants are frequently used to wrap nerves for scarring prevention, but not as nerve conduits, they will be listed in a separate subgroup in this review. Looking at the underlying material origin, the spacer materials described in the examined literature can be divided into four different (sub-)groups, as shown in Table 3. These include tissue-derived materials, protein-based materials and polysaccharide materials, which are all counted as natural materials, and materials based on synthetic polymers.

*Tissue-derived materials:* Baltu et al. described an improved epineural scar density score after autologous buccal mucosa graft application [27]. After applying free fat grafts from the rat’s groin region, Dumanian et al. showed a decreased nerve stiffness following epineurectomy [13].

Furthermore, allogenic vein transplants have been thoroughly investigated as a nerve wrap beforehand, and Murakami et al. showed reduced epineural scarring in descriptive histology [22]. Özgenel et al. investigated the use of a human amniotic membrane xenograft and found improved macroscopic adhesion scores after epineurectomy [20]. In another group, Özgenel et al. combined the human amniotic membrane with 1% hyaluronic acid (H.A.) and observed improved results in the adhesion scores and the scar thickness measurement [20].

*Protein-based materials:* Using collagen fibers to wrap the nerve and compare the effects of the proteinase inhibitor aprotinin to phosphate-buffered saline (PBS), Görgülü et al. found improved adhesion scores in the aprotinin group, but not in the PBS group [14]. ADCON-T/N is a gel composed of porcine gelatin and a polyglycan ester in PBS. The group treated with the ADCON-T/N gel was observed to have less macroscopic scar formation than both, the untreated control group as well as another group treated with a control gel, described by the authors as lacking a specific carbohydrate component [15,30]. While this, as many others, represents a hybrid between different categories, it was listed in this category because of gelatin’s main component, collagen.

*Polysaccharide-based materials:* Hyaluronic acid gel is part of eight investigated groups of the included studies, representing the most-used material in this cohort [17,18,19,20,23,25]. While it was investigated in combination with other materials in three groups, it was also tested on its own in five groups, showing a positive effect in four of these [17,18,19]. As mentioned above, it is described to have positive results in combination with a human amniotic membrane graft [20]. Another combination is hyaluronic acid with a chitosan conduit, showing a lower scar collagen density than the control group as described by Li et al. [23]. In this study, the chitosan conduit is also investigated on its own. While both the chitosan conduit and the HA show positive influences when applied individually, the best results are achieved after combining both [23]. As the only included study describing no improved results in any intervention group after spacer application, Hernández-Cortés et al. tested the effects of oxidized regenerated cellulose and evaluated scar formation using quantitative connective tissue measurement [25]. Ohsumi et al. examined a viscous alginate sol as a spacer material, which by resulting in a lower biomechanical breaking strength demonstrated scarring prevention, further supported by descriptive histology [21]. Two gels of different viscosities made by combining Carboxymethylcellulose (CMC) with the phosphoglyceride phosphatidylethanolamine (PE) were investigated by Yamamoto et al. and demonstrated to have positive effects in both versions. However, the group treated with the lower viscosity gel showed less scarring in the macroscopic evaluation and in breaking strength testing [17].

*Materials based on synthetic polymers:* Finsterbush et al. investigated the use of a longitudinally cut silicone tube, showing lower scar tissue formation than the control group in descriptive histology [26]. Three additional studies examined the effects of polylactide (PLA) in different forms [16,19,24]. Kikuchi et al. presented superior effects of E8002, a PLA-based membrane with ascorbic acid, over the control group in adhesion scores and optical scar density, but the same membrane without ascorbic acid did not show improved outcomes [24]. A PLA film with a honeycomb texture demonstrated better results in descriptive histological and adhesion strength analysis, which was not the case for the same film in a cast texture as examined by Okui et al. [16]. Shintani et al. found improved adhesion scores, biomechanical breaking strength, and descriptive histological results after applying a conduit made of PLA and poly(ε-caprolactone) [19].

## 4. Discussion

Of 28 included intervention groups, 21 (75%) demonstrated a preventive effect on scar development after spacer material application in at least one qualitative or quantitative described endpoint. With 8 different scar induction methods and various outcome measures used to assess the effect of 23 different spacer materials, the overall heterogeneity of the described sample is high. Even though inclusion criteria for this systematic review were set tightly to increase comparability, a quantitative analysis was not possible.

Overall, the enormous efforts in testing a variety of used materials suggest that the technique of spacer material application around peripheral nerves to prevent perineural adhesions is promising, and results might be attributable to the technique as well as to the used material. Nerve wrapping might be a feasible technique to prevent extensive adhesion and scar formation after peripheral nerve injury. However, the current literature does not allow a conclusion on the relative significance of the material selection compared to the independent impact of the surgical technique itself. Nevertheless, feasible wrapping materials need to have certain basic properties to be suited for this indication. Biocompatibility plays a key role, and in future clinical settings, different aspects of patient-personalized material selection must be considered. The goal is to provide an effective mechanical barrier against adhesion formation between the nerve and the paraneural tissue and at the same time create an optimal environment for nerve perfusion, mobility, and nutrition and, thus, nerve regeneration [29].

Biocompatibility may vary from the animal model to the human model. Some materials, like silicone, might show favorable outcomes in animal studies with a limited follow-up, but lead to complications in clinical application [26,31,32]. Adverse reactions, as observed in some materials, run contrary to the goals of placement [31,33,34]. Furthermore, the size and diameter of the wrapping material are essential to avoid iatrogenic constriction or overfitting, possibly reducing the desired effects [35,36].

Tissue-based spacer materials were used in four of the included intervention groups [13,20,22,27]. Nerve wraps based on tissue and nerve covering techniques using transplanted tissue have been researched for decades, with cases of clinical application reported for different grafts and flaps [9,37,38,39,40,41]. Additional soft tissue coverage or wrapping with compatible tissues, like veins, shows promising results and is current practice as a last resort in clinical practice [8]. Their use comes with advantages including high biocompatibility, favorable biodegradation and no additional purchasing costs in the case of autologous transplants. Especially the vein graft has been well-researched from different perspectives in pre-clinical and clinical models [9,22,42,43,44,45,46,47,48,49]. Human amniotic membrane wrapping is described as an option in treatment for recurrent compression neuropathies due to its high biocompatibility and previously reported success in scar prevention [50,51]. Fat grafts or flaps are described as a successful salvage option in recurrent compression neuropathies [37,38,41]. While buccal mucosa has not been researched in larger-scale clinical application studies, it generally fulfills the criteria of biocompatibility and glide apparatus preservation. In general, tissue-based materials are well-researched in peripheral nerve surgery and frequently achieve significantly improved scar prevention and treatment results; some are already in clinical use. On the other hand, depending on the exact material, several aspects, including origin, donor site morbidity, availability, cost, and potential adverse reactions, must be considered individually for each patient [33]. While veins are frequently used due to their easy grafting and limited donor site morbidity, it is unclear if one of these materials produces superior outcomes regarding scarring and adhesion prevention.

Four groups used protein-based spacer materials to prevent peripheral nerve scarring, including collagen, cellulose, and gelatin [14,15,25]. Collagen has previously been described in clinical applications for nerve wrapping after peripheral nerve surgery [52,53,54,55]. Next to promising results in clinical testing, animal models have confirmed several positive effects of collagen, including immunomodulation [56], neuroregeneration [47,57,58,59], and pain alleviation after peripheral nerve injury [53,60]. Several collagen-based nerve wraps and conduits are available on the market and approved for in-patient use, bringing this technique from bench to bedside. Petersen et al. tested the effect of ADCON-T/N gel and the effect of a control gel without one specific carbohydrate component [15]. The basis for this gel is gelatin, largely composed of collagen, combined with a polyglycan ester in PBS [30]. While the control gel did not show scar preventive effects, ADCON gel has been demonstrated to have preventive effects on adhesion formation in tendon injuries and spinal peridural fibrosis additionally to peripheral nerves [15,30,61]. However, case reports of patients experiencing cerebrospinal fluid leaks after the use of the ADCON gel led to the suspension of its use in spinal surgery [62]. This is one example of materials that produce favorable outcomes in an experimental setting but might not be suitable for frequent clinical use, as conditions in humans differ.

The group of polysaccharide-based wrapping materials includes five used materials in the investigated studies. Although Hernández-Cortés et al. did not show an effect in preventing perineural adhesions using a cellulose-based wrapping material [25], cellulose has been examined with positive results in other research, including use around nerves and tendons to prevent adhesions [63,64,65]. Improved oxygen and glucose diffusion through cellulose conduits to nerves has been demonstrated [66,67]. Supporting the promising literature, Yamamoto et al. showed perineural adhesion prevention after using a CMC-PE gel [17]. Similarly, Urano et al. demonstrated improved neuroregeneration after CMC-PE application in an animal model of chronic nerve compression [68]. Hyaluronic acid gel is used in four of the included studies and shows scar preventive effects in three of them [17,18,19,23]. It has been described as a scar-preventive agent on its own after peripheral nerve injuries in previous studies [69,70]. Hyaluronic acid gel seems to show the best results when combined with other methods, as Li et al., Özgenel et al., and others have shown [20,23,71,72,73]. Another representative of the polysaccharide group is alginate, which is already under promising investigation as both a hydrogel and a conduit material in peripheral nerve research [74]. Due to its biochemical and biomechanical properties, it works well as a mechanical barrier to adhesion formation [74]. Last, chitosan, a polysaccharide naturally found in arthropod shells, has been broadly researched in peripheral nerve surgery [75]. Li et al. described its scar-preventive effects on its own and combined with hyaluronic acid [23]. As a conduit and in crystalline form, chitosan prevents epineural scar formation [76]. Its immunomodulatory properties seem to create a favorable regenerative environment after peripheral nerve injury. Therefore, chitosan was investigated clinically in its use to protect coaptation sites of peripheral nerves [75,77].

While silicone was described in earlier studies to prevent perineural scar formation and improve neural regeneration, its use has increasingly become unpopular [26,78]. Although silicone is biocompatible, its non-biodegradability poses clinical use problems, potentially leading to increased fibrosis [31,34]. Today, silastic tube cuffing is even used as a scarring induction method in experimental models [11]. Contrarily, polylactide generally is well-biodegradable [79]. Okui et al. investigate it in a honeycomb and a cast morphology, reporting superior results in the honeycomb and frequent dislocation in the cast morphology [16]. It can be modified using different other materials, as described by investigated studies with poly(ε-caprolactone) or ascorbic acid [19,24]. Clinically, adverse reactions, including inflammatory responses and delayed biodegradation, have been mentioned in combination with PLA- poly(ε-caprolactone) [34]. Ascorbic acid, on the other hand, has been preclinically observed to accelerate Wallerian degeneration and improve neural regeneration [80,81].

As indicated by the positive effect of the described studies, nerve wrapping appears to have a preventive effect on scar and adhesion formation around peripheral nerves. However, it remains unclear which materials are optimal for this purpose. While the concept per se seems to have the desired effect, further studies are needed in order to elaborate on the strengths and weaknesses of different materials. While some materials are already approved for clinical use, future comparative research needs to be conducted on their effects in order to optimize their indication in the sense of personalized treatments based on each patient’s individual case [10].

As illustrated by some of the investigated materials, biocompatibility is essential for the safe application of nerve-wrapping materials, and long-term effects need to be explored before widespread clinical use [10,15,26,31,34,62]. Attention should also be brought to the intraoperative technique of applying nerve wraps. If they are wrapped around the nerve too tightly, this can lead to adverse effects creating increased scar formation and hindering nerve regeneration [82].

Besides nerve wrapping, various essentially different approaches for the prevention of perineural adhesion formation have been explored. These range from local and systemic pharmaceutic interventions, like intraperitoneal verapamil injection [83], over cellular applications to external radiation and frequently show promising results [84,85,86,87]. However, the sheer quantity of research on wrapping spacers and mostly positive results indicates that the effect of wrapping the nerve should not be underestimated. From a practical viewpoint, the success of external radiation and repetitive pharmaceutical interventions, both described as scar-prevention methods, are highly patient-dependent and not easy or economical to administer. Considering the enormous amount of tested spacer materials, refining the spacer technique will be an increasingly big part of the research process in the future.

Görgülü et al. described the combination of collagen fibers as a wrapping material in combination with aprotinin acting as a pharmaceutic anti-scarring agent [14]. The combination of wrapping materials with bioactive agents has been used to improve peripheral nerve regeneration and prevent nerve adhesions in research. This includes drugs limiting fibroblast activity and suppressing inflammatory responses [83,86,87,88,89]. Furthermore, different bioactive agents, including drugs and mesenchymal stem cells, can be used to improve nerve regeneration and create a favorable environment for neurite outgrowth [28,90,91,92]. Four studies included in this systematic review compared hybrid models, adding different agents to wrapping materials, with the use of only one of the components [14,20,23,24]. Favorable results in using these hybrid models suggest that they will be a future refinement enhancing the positive effects of spacer application for the prevention of peripheral nerve adhesions.

In this study, several limitations exist: (1) by excluding transection injuries to homogenize results, potential candidates were left out; (2) the heterogeneity of scarring induction and assessment methods makes direct comparisons barely possible; and (3) all investigated studies use the rat sciatic nerve model which is frequently used. However, it is not optimal in mimicking human peripheral nerve physiology and pathology [93,94]. Therefore, the direct translation of positive effects from the animal model to humans can be limited and requires thorough investigation before the clinical implementation of wrapping materials in patients. Mainly, the heterogeneity in injury types and, in some cases, the lack of distinction between primarily aiming for improved nerve regeneration or decreased scarring introduces difficulties in the comparison of results and thus slows a transition to systematic clinical testing.

## 5. Conclusions

Our systematic review of different methods for peripheral nerve wrapping demonstrates that most of the literature describes positive effects on preventing peripheral nerve adhesions and scarring by applying a biocompatible spacer material in the animal model. The existing wrapping materials have to be evaluated using standardized and comparative animal models to filter out the most promising candidates before a transition from bench to bedside can be made.

## Figures and Tables

**Table 1:** Basic study characteristics of all included studies and their intervention groups. Multiple intervention groups within the same study are labelled with letters A–D. For commercially produced wrappers, the production company is depicted in brackets behind the material. Abbr.: PBS = phosphate-buffered saline, AA = ascorbic acid, HA = hyaluronic acid, PLA = polylactide, PCL = poly(ε-caprolactone), CMC = carboxymethylcellulose (CMC), PE = phosphatidylethanolamine, n = number of animals in group, FU = follow-up time in weeks.

Author	Year	Group	Scarring Induction	Wrapping Material	n	FU
Baltu et al. [27]	2017	A	Epineurectomy	Buccal mucosa graft	24	8
Dumanian et al. [13]	1999	A	Epineurectomy	Free fat grafts	14	8
Finsterbush et al. [26]	1982	A	Crush injury, muscle cauterization	Semirigid 15 mm silicone tube, cut longitudinally	30	8
Görgülü et al. [14]	1998	A	External neurolysis	Collagen fibers soaked with aprotinin	22	6
		B	Abrasive injury	Collagen fibers soaked with aprotinin	22	6
		C	External neurolysis	Collagen fibers soaked with PBS	22	6
		D	Abrasive injury	Collagen fibers soaked with PBS	22	6
Hernandez-Cortes et al. [25]	2010	A	Perineurectomy + muscle cauterization	Oxidized regenerated cellulose wrap	40	6
Kikuchi et al. [24]	2020	A	Muscle cauterization	E8002 wrapping (Kawasumi Laboratories Inc., Tokyo, Japan)	7	6
		B	Muscle cauterization	E8002 AA- wrapping (Kawasumi Laboratories Inc., Tokyo, Japan)	7	6
Li et al. [23]	2018	A	Crush injury	Chitosan conduit	15	12
		B	Crush injury	HA gel	15	12
		C	Crush injury	Chitosan conduit + HA gel	15	12
Murakami et al. [22]	2014	A	Chronic constriction injury by nerve ligation	Allogenic vein wrap	30	20
Ohsumi et al. [21]	2005	A	External/internal neurolysis + muscle cauterization	Viscous alginate sol	8	6
Okui et al. et al. [16]	2010	A	Internal neurolysis + muscle cauterization	Honeycomb poly-lactide film	42	6
		B	Internal neurolysis + muscle cauterization	Cast poly-lactide film	12	6
Özgenel et al. [20]	2004	A	Epineurectomy	Human amniotic membrane	12	12
		B	Epineurectomy	Human amniotic membrane + HA injection	12	12
Petersen et al. [15]	1996	A	Internal neurolysis	ADCON-T/N gel (Gliatech, Inc., Cleveland, OH, USA)	9	4
		B	Internal neurolysis	Control gel	9	4
Shintani et al. [19]	2018	A	Muscle cauterization	PLA/PCL tube	12	6
		B	Muscle cauterization	1% HA	8	6
Smit et al. [18]	2004	A	External neurolysis	1% HA	5	6
		B	Crush injury	1% HA	7	6
Yamamoto et al. [17]	2010	A	Internal neurolysis	1% HA	18	6
		B	Internal neurolysis	CMC-PE hydrogel, low viscosity	18	6
		C	Internal neurolysis	CMC-PE hydrogel, high viscosity	18	6

**Table 2:** Scarring induction and assessment methods of all included studies and their intervention groups. Multiple intervention groups within the same study are labelled with letters A–D. For commercially produced wrappers, the production company is depicted in brackets behind the material. The right two columns show the outcomes as assessed in the study with quantitative or descriptive assessment methods. Abbr.: PBS = phosphate-buffered saline, AA = ascorbic acid, HA = hyaluronic acid, PLA = polylactide, PCL = poly(ε-caprolactone), CMC = carboxymethylcellulose (CMC), PE = phosphatidylethanolamine.

Author	Year	Group	Scarring Induction	Wrapping Material	Scar Assessment Method	Scar Prevention Quantitative Ass.	Scar Prevention Descriptive Ass.
Baltu et al. [27]	2017	A	Epineurectomy	Buccal mucosa graft	Adhesion score (Petersen et al. 1996 [15]), epineural scar density score	yes	yes
Dumanian et al. [13]	1999	A	Epineurectomy	Free fat grafts	Nerve stiffness	yes	yes
Finsterbush et al. [26]	1982	A	Crush injury, muscle cauterization	Semirigid 15 mm silicone tube, cut longitudinally	Desriptive histology	not described	yes
Görgülü et al. [14]	1998	A	External neurolysis	Collagen fibers soaked with aprotinin	Adhesion score (Petersen et al. 1996 [15])	yes	yes
		B	Abrasive injury	Collagen fibers soaked with aprotinin	Adhesion score (Petersen et al. 1996 [15])	yes	yes
		C	External neurolysis	Collagen fibers soaked with PBS	Adhesion score (Petersen et al. 1996 [15])	no	no
		D	Abrasive injury	Collagen fibers soaked with PBS	Adhesion score (Petersen et al. 1996 [15])	no	no
Hernandez-Cortes et al. [25]	2010	A	Perineurectomy + muscle cauterization	Oxidized regenerated cellulose wrap	Connective tissue measurement	no	no
Kikuchi et al. [24]	2020	A	Muscle cauterization	E8002 wrapping (Kawasumi Laboratories Inc.)	Adhesion score (Petersen et al. 1996 [15]), optical scar density	yes	yes
		B	Muscle cauterization	E8002 AA- wrapping (Kawasumi Laboratories Inc.)	Adhesion score (Petersen et al. 1996 [15]), optical scar density	no	no
Li et al. [23]	2018	A	Crush injury	Chitosan conduit	Adhesion score (Petersen et al. 1996 [15]), epineurium collagen density	yes	yes
		B	Crush injury	HA gel	Adhesion score (Petersen et al. 1996 [15]), epineurium collagen density	yes	yes
		C	Crush injury	Chitosan conduit + HA gel	Adhesion score (Petersen et al. 1996 [15]), epineurium collagen density	yes	yes
Murakami et al. [22]	2014	A	Chronic constriction injury by nerve ligation	Allogenic vein wrap	Descriptive histology	not described	yes
Ohsumi et al. [21]	2005	A	External/internal neurolysis + muscle cauterization	Viscous alginate sol	Biomechanical breaking strength, descriptive histology	yes	yes
Okui et al. et al. [16]	2010	A	Internal neurolysis + muscle cauterization	Honeycomb poly-lactide film	Descriptive histology, descriptive macroscopic adhesion strength	not described	yes
		B	Internal neurolysis + muscle cauterization	Cast poly-lactide film	Descriptive macroscopic adhesion strength	not described	no
Özgenel et al. [20]	2004	A	Epineurectomy	Human amniotic membrane	Adhesion score (Petersen et al. 1996 [15]), scar thickness measurement	yes	yes
		B	Epineurectomy	Human amniotic membrane + HA injection	Adhesion score (Petersen et al. 1996 [15]), scar thickness measurement	yes	yes
Petersen et al. [15]	1996	A	Internal neurolysis	ADCON-T/N gel (Gliatech, Inc., Cleveland, OH, USA)	Adhesion score (Petersen et al. 1996 [15]), scar area measurement	yes	yes
		B	Internal neurolysis	Control gel	Adhesion score (Petersen et al. 1996 [15]), scar area measurement	no	no
Shintani et al. [19]	2018	A	Muscle cauterization	PLA/PCL tube	Adhesion score (Petersen et al. 1996 [15]), biomechanical breaking strength, descriptive histology	yes	yes
		B	Muscle cauterization	1% HA	Adhesion score (Petersen et al. 1996 [15]), biomechanical breaking strength, descriptive histology	no	no
Smit et al. [18]	2004	A	External neurolysis	1% HA	Biomechanical breaking strength	yes	yes
		B	Crush injury	1% HA	Biomechanical breaking strength	yes	yes
Yamamoto et al. [17]	2010	A	Internal neurolysis	1% HA	Adhesion score, biomechanical breaking strength, descriptive scar area measurement	no	yes
		B	Internal neurolysis	CMC-PE hydrogel, low viscosity	Adhesion score, biomechanical breaking strength, descriptive scar area measurement	yes	yes
		C	Internal neurolysis	CMC-PE hydrogel, high viscosity	Adhesion score, biomechanical breaking strength, descriptive scar area measurement	yes	yes

**Table 3:** Used biomaterials and their according groups. Abbr.: PBS = phosphate-buffered saline, PLA = polylactide, PCL = poly(ε-caprolactone), CMC = carboxymethylcellulose (CMC), PE = phosphatidylethanolamine.

Biomaterial	Material Type	Origin
Buccal mucosa graft	Tissue-based	Natural
Fat graft		
Vein graft		
Human amniotic membrane		
Collagen fibers + aprotinin	Protein-based	
Collagen fibers + PBS		
ADCON-T/N gel (gel composed of gelatin and a carbohydrate polymer in PBS)		
ADCON-T/N control gel		
Oxidized regenerated cellulose wrap	Polysaccharide-based	
CMC-PE hydrogel		
Hyaluronic acid (gel)		
Alginate sol		
Chitosan		
Silicone	Synthetic Polymer	Synthetic
PLA		
PLA-PCL		
E8002 (PLA-based membrane with L-ascorbic acid)		

## Data Availability

Not applicable.
